# The antibacterial effect of nanosilver fluoride in relation to caries activity in primary teeth: a protocol for a randomized controlled clinical trial

**DOI:** 10.1186/s13063-022-06477-5

**Published:** 2022-07-08

**Authors:** Nour Ammar, Magda M. El-Tekeya, Sara Essa, Marwa M. Essawy, Samar N. El Achy, Dalia M. Talaat

**Affiliations:** 1 Department of Pediatric Dentistry and Dental Public Health, Faculty of Dentistry, Alexandria University, Alexandria, Egypt; 2 Department of Medical Microbiology and Immunology, Faculty of Medicine, Alexandria University, Alexandria, Egypt; 3 Department of Oral Pathology, Faculty of Dentistry, Alexandria University, Alexandria, Egypt; 4 Center of Excellence for Research in Regenerative Medicine and Applications (CERRMA), Faculty of Medicine, Alexandria University, Alexandria, Egypt; 5 Department of Pathology, Faculty of Medicine, Alexandria University, Alexandria, Egypt

**Keywords:** Nanosilver fluoride, Silver diamine fluoride, Antibacterial, Caries, Microbiology, Saliva, Primary teeth, Pediatric dentistry, Clinical trial

## Abstract

**Background:**

Minimally invasive dentistry is a highly convenient and efficient method of managing caries in pediatric patients. Silver diamine fluoride (SDF) is commonly used to arrest active caries lesions. However, the associated black stain, possibility of soft tissue injury, and unpleasant taste often limit its use. Recently, nanosilver fluoride (NSF) emerged as a promising topical fluoride agent with potent cariostatic and antibacterial potentials. This novel anticaries agent has gained attention as an alternative to overcome the drawbacks of SDF in caries arrest.

**Objectives:**

To assess the antibacterial effect of NSF in relation to caries activity in dentin caries lesions, as well as to investigate the change in saliva bacterial levels in primary teeth in comparison to SDF after 1 and 3 months.

**Materials and methods:**

Fifty children aged 4 to 6 years old with active dentin caries lesions (score 5 according to International Detection and Assessment System (ICDAS II) criteria) will be enrolled in the study. They will be equally and randomly allocated into 2 groups: a group receiving NSF and a control group receiving SDF treatment. Microbiological samples will be collected from the carious lesions and from unstimulated saliva at the baseline and at the 1 and 3 months’ follow-up appointments. Bacterial counts will be assessed using Mitis Salivarius agar (selective culture media for S. mutans) and Rogosa agar (selective culture media for lactobacilli), and the results will be expressed in colony-forming units. Data regarding the children’s oral health will be collected and their dmf index will be scored. The arrest of active carious lesions will be measured at the follow-up appointments according to ICDAS II criteria.

**Results:**

The relation between bacterial colony counts and lesion activity for both groups will be assessed, as well as the change in salivary bacterial counts. The collected data will be statistically evaluated and tabulated. This clinical trial has been registered on ClinicalTrials.gov in January 2022 (original version) with ID: NCT05221749.

**Supplementary Information:**

The online version contains supplementary material available at 10.1186/s13063-022-06477-5.

## Introduction

Dental caries is a chronic disease, ranking among the most highly prevalent diseases in children [1]. Minimally invasive approaches to prevent and arrest caries lesions provide methods to address the caries problem that are affordable and convenient and do not require sophisticated equipment [2]. Silver diamine fluoride (SDF) has a proven antibacterial action [3] and assists enamel remineralization [4], and is available for arresting caries as a simple, non-invasive medication [5, 6]. Its major drawback is the black staining of carious tissue and that it may induce reversible slightly painful lesions in the oral mucosa that usually disappear within 48 h [7]. Moreover, it has a reported unpleasant taste, and there are concerns about toxicity [7, 8].

The use of nanotechnology in dentistry has recently attracted significant attention [9], specifically, formulations of silver nanoparticles (AgNPs). Nanosilver fluoride (NSF) is a yellow solution containing uniformly dispersed AgNPs and fluoride. This new agent is safe to be used in humans [10, 11] and has several desirable properties. Multiple in vitro researches have investigated the antimicrobial effects of AgNPs on carious tooth structures and have confirmed the substantial antibacterial effect of NSF on *Streptococcus mutans* and *Lactobacilli* [12–16]. Additionally, several studies [10, 17, 18] have reported that NSF is effective in arresting active dentine caries without any distinctive tissue pigmentation.

To our knowledge, no publication has reported the in vivo antimicrobial efficiency of NSF on the primary dentition in comparison to SDF. Therefore, this study will be conducted to evaluate and compare the antibacterial and caries activity of NSF versus SDF in dentin caries lesions and saliva in primary teeth. Specifically, in active lesions have a distinct cavity with visibly exposed dentine, where the cavity feels soft or leathery on gentle probing (known as active score 5 ICDAS II lesions). The null hypothesis of this study is that no statistically significant difference will be detected between NSF and SDF by comparing their antibacterial effect in relation to caries activity in dentin caries lesions, as well as that there will be no statistically significant difference in the change in salivary bacterial levels after 1 and 3 months of follow-up.

## Materials and methods

### Study design

**Fig. 1:**
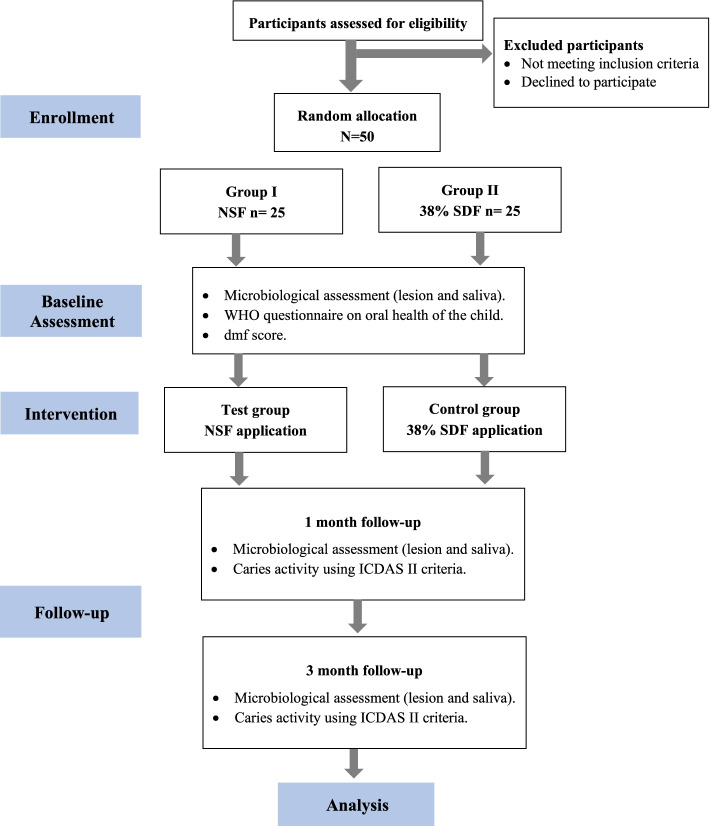
Study plan flowchart

**Fig. 2:**
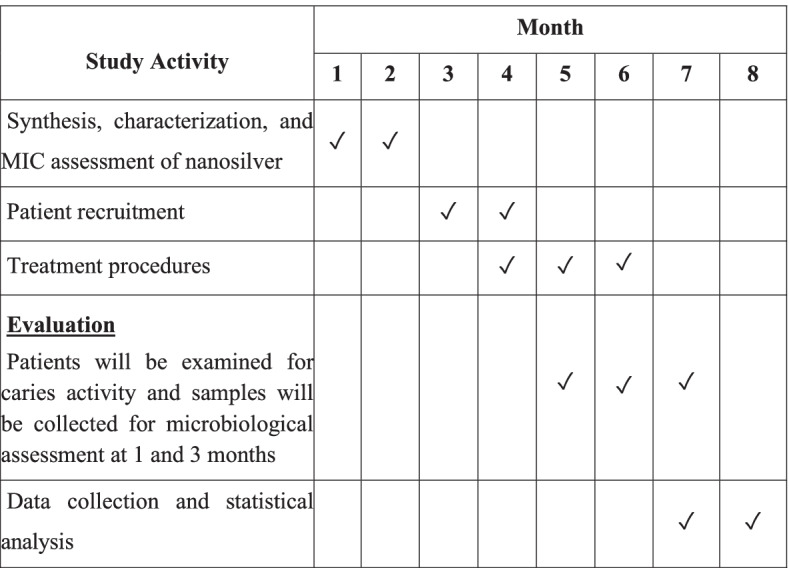
Study timeline

This will be a parallel, two-arm, equivalence randomized controlled clinical trial, with a 1:1 allocation ratio. It will be set up and reported according to the CONSORT guidelines [19]. It has been approved by the ethics committee of the Faculty of Dentistry, Alexandria University IRB NO: 00010556 – IORG 0008839 and been registered on ClinicalTrials.gov in January 2022 with ID: NCT05221749, https://clinicaltrials.gov/ct2/show/NCT05221749. The detailed procedures that shall be taken in ethics consideration are mentioned in the supplementary material. The study plan is demonstrated in Fig. 1, and the study timeline is demonstrated in Fig. 2.

The PICOT question is: will children (4–6 years old) with an active carious lesion (ICDAS score 5) (population; P) using nanosilver fluoride varnish (Intervention; I) in comparison to silver diamine fluoride (comparison; C) show a greater percentage decrease in bacterial counts (outcome; O) after 3-month follow-up (time; T)?

### Study setting and location

The study will be conducted in the Department of Pediatric Dentistry and Dental Public Health of the Faculty of Dentistry and the Department of Medical Microbiology and Immunology of the Faculty of Medicine. The nanosilver fluoride will be prepared and characterized at the Center of Excellence for Research in Regenerative Medicine and Applications (CERRMA), Faculty of Medicine, Alexandria University, Alexandria, Egypt.

### Sample size estimation

Sample size was estimated assuming 5% alpha error and 80% study power. According to Dos Santos et al. [3], after 5 months, the NSF group had 72.7% arrested decay. Therefore, it was estimated that 14.54% of lesions treated with NSF will be arrested after 1 month. According to Milgrom et al. [3], 51.7% of lesions treated with 38% silver diamine fluoride (SDF) have been arrested after 21 days. Based on difference between two independent proportions, the minimum sample size was calculated to be 24 patients per group, increased to 25 teeth to make up for lost to follow-up cases. Total sample size = number per group × number of groups = 25 × 2 = 50 patients. The sample size estimation was based on Rosner’s method [20] and calculated using G*Power 3.1.9.7 [21].

### Eligibility criteria

The participants to be enrolled in this study will be recruited after fulfilling the following criteria:

#### Inclusion criteria

- Children 4–6 years old.
- The presence of at least one active carious lesion on a primary tooth, with a score of 5 according to the International Detection and Assessment System (ICDAS II), detected by visual-tactile inspection to assess lesion severity and activity [22] (Appendix 2, Tables 1 and 2).
- Completion of an informed consent to participate in the study (Appendix 1).

#### Exclusion criteria

- Children reporting spontaneous or elicited pain from caries or showing any signs of pulpal infection, swelling, abscess, obvious discoloration of the tooth, or premature mobility [7].
- Reported usage of local or systemic antibiotics, chlorhexidine, or fluoride mouthwashes within the last 2 weeks [23].
- Children who have special health care needs or who are undergoing medical treatment for chronic or acute diseases that affect salivary flow.
- Allergy or sensitivity to silver or any of the materials included in the study [21].
- Child weight less than 10 Kg (to avoid concerns for toxicity) [24].

### Randomization and allocation

Subjects complying with the inclusion criteria will be randomly assigned using a computer-generated list of random numbers to either the NSF or the SDF group [25]. Allocation will be performed by a trial independent individual and the allocation ratio is intended to be equal (1:1). Participants will be randomly and equally allocated to one of the two treatment groups:

- Intervention group (NSF): Twenty-five children meeting the eligibility criteria will receive nanosilver fluoride.
- Control group (SDF): Twenty-five children meeting the eligibility criteria will receive 38% silver diamine fluoride.

### Allocation concealment

An assistant will be responsible for giving each participant a serial number that will be used for his/her allocation. A duplicate of this number will be kept in an opaque envelope indicating to which group the patient belongs. This envelope will be kept by a trial independent individual who will be assigned the role of opening it only at the time of intervention; so that the group to which the child is allocated is concealed from the investigator [26]**.** The investigator will not be blinded to the treatment type as the SDF solution has a bluish tint and will stain the active caries lesions black, while the NSF solution has a yellowish tint and is not expected to stain the lesions. However, the participants, statistician, and microbiologist will be blinded to the treatment group.

### Training, calibration, and reliability

The principal investigator will be trained and calibrated by the study supervisors. Training and calibration will be regarding the use of ICDAS II criteria in the diagnosis of the teeth to be included in the study [27], application of both test materials [28], and evaluation of outcomes. The examination of 10 participants meeting the eligibility criteria will be carried out followed by re-examination after 7 days for the determination of intra-examiner agreement measured by Cohen’s kappa (Κ). Obtaining a kappa score ≥0.8 is considered excellent reliability. These participants will not be included in the study sample.

### Nanosilver fluoride preparation

#### Synthesis and characterization of silver nanoparticles

In a 250-ml beaker, 8 mg of silver nitrate (Sigma-Aldrich, US) will be added to 50 mL of deionized water containing 0.5% (v/v) polyethylene glycol (PEG 400, Sigma-Aldrich, US). The solution will be heated until the boiling point, then 1 mL containing 50.5 mg of trisodium citrate (Alpha Chemika, India) will be added. Change in the color of the solution to yellow will indicate the formation of AgNPs [29].

The preliminary detection of synthesized AgNPs will be carried out using a UV–visible spectrophotometer (Nanodrop, DeNovix, DS-11 FX+, US) by scanning the absorbance spectra in the range of 200–800 nm wavelength. The average particle size, polydispersity index (PDI), and particle charge of AgNPs will be performed by the dynamic light scattering technique using Zeta-seizer (Nano ZS, Malvern Instruments, Worcestershire, UK), with a dilution ratio of 1:6 [30], in addition to transmission electron microscopy (TEM) (JEOL JEM-1400 series 120kV TEM).

#### Cytotoxicity assay

The safety profile of the synthesized PEG-nanosilver will be investigated on human gingival fibroblasts to ensure its safety. All cultivating materials [Dulbecco’s modified Eagle’s medium (DMEM), fetal bovine serum (FBS), phosphate-buffered saline (PBS), and antibiotics (penicillin and streptomycin)] will be obtained from Gibco, US. Reagents of cytotoxicity assay as MTT 3-(4, 5-dimethythiazol-2-yl)-2, 5-diphenyltetrazolium bromide will be purchased from SERVA-Electrophoresis GmbH, Germany, while dimethyl sulfoxide (DMSO) will be obtained from Fisher Chemical (Fisher Scientific), UK.

The cytotoxic effect of PEG-coated AgNPs on the viability of the gingival fibroblasts cells will be evaluated using MTT assay at 24 h incubation period. The cells from the primary culture will be seeded onto a 96-well culture plate containing 100 μL of DMEM/well at a density of 7000 cells/well. The cells will be incubated for 24 h at 37 °C in 5% CO_2_. The gingival fibroblasts will then be treated with serial concentrations ranging from 10 to 1000 μM of PEG-AgNPs. Untreated cells will be used as control.

After 24 h incubation, the cells will be washed twice with PBS and then 100 μL of MTT (0.5 mg/mL DMEM) will be added to each well and incubated at 37 °C for 3–4h. Thereafter, the medium containing MTT will be removed, and the cells will be lysed by adding 100 μL /well DMSO in darkness. Then, an ELISA reader will quantify the optical density (OD) of the dissolved formazan crystals at 570 nm using a micro plate scanning spectrophotometer (ELISA reader, Infinite F15 TECAN, Switzerland). The percentage of cell viability will be expressed as mean ± SD according to the following formula: Viable cell % = (OD treated / OD untreated) × 100, where “OD treated” represents the absorbance value of the treated sample and “OD untreated” represents the absorbance value of the corresponding untreated sample [31]. The cytotoxicity assay will be performed in triplicates for 3 independent experiments.

#### Determination of MIC of silver nanoparticles

The MIC (mean inhibitory concentration) of PEG-AgNPs will be determined by visual comparison of the differences in turbidity in a 96-well plate [32]. *Streptococcus mutans* (proficiency testing strain identified by the College of American Pathologists as *S. mutans*) will be prepared in brain heart infusion with 2% sucrose for a concentration of 0.5 on the McFarland scale, and this will be verified using colorimetric measurement (Vitek, bioMérieux, Mexico City, Mexico). This concentration is equivalent to 1.5 × 10^8^ CFU/mL. The MIC of PEG-AgNPs solution will be determined with ranges of 0.25–1024 mg/L in double fold dilutions prepared according to the Clinical and Laboratory Standards Institute (CLSI) broth microdilution method [33].

In microdilution plates, 100 μL of the dispersion form of AgNPs will be placed in wells with predetermined serial dilutions, and then 100 μL brain–heart infusion with 2% sucrose will be inoculated onto the wells with *S. mutans* at 1 × 10^6^ CFU/mL. The first and last columns of the plates will be used as positive and negative controls, respectively. Finally, the plates will be incubated at 37 °C for 24 h. Determination of the MIC will be done based on turbidity.

#### Functionalization of silver nanoparticles with sodium fluoride

For fluoridation of the formulated AgNPs, 22,600 ppm of sodium fluoride will be dissolved in 10 ml of PEG-AgNPs at the determined MIC. The fluoridated nanosuspension will then be left under stirring, in a light proof brown bottle, overnight, at room temperature [17].

### Treatment protocol

#### Effect modification/confounders

The socioeconomic level, dietary, and oral health practices of children (as reported by their caregivers) will be measured at baseline using the World Health Organization’s questionnaire for assessment of children’s oral hygiene habits (Appendix 3) [34]. The participants will be provided the needed dental treatments before the beginning of the study period and after its completion. This is to avoid having any confounding factors (new restorations, extractions, topical fluoride treatments) that may cause an unaccounted change in the microbiological levels before and after the interventions. This is to ensure that the change in bacterial level is mainly brought about by the intervention groups and to provide an outcome measurement that is as accurate as possible.

##### Baseline examination

After obtaining the informed consent from the care giver/parent, the researcher (NA) will provide oral hygiene instructions to each of the study participants and will educate them about the importance of maintaining good oral hygiene and a proper diet [35]. The patients and caregivers will be informed about the therapeutic intervention to be applied, the possible outcomes, and side effects in detail. All the examinations and interventions will be done by one calibrated examiner. All of the lesions in the children’s oral cavity indicated for treatment with NSF or SDF will be treated and sampled for microbiological analysis [28]. No more than one drop of SDF or NSF shall be used in one appointment to avoid concerns for toxicity [24, 28].

On the day of the intervention, the children will be asked to refrain from tooth brushing in the morning, as well as eating and drinking (except water) for at least 2 h before the appointment. The dmf index will be recorded for each patient. For caries treatment in all groups, no caries or unsupported enamel will be removed. Unstimulated whole saliva samples will be first collected by asking the patient to drool in a collection cup until at least 1 mL of saliva is collected [36]. Then any gross debris surrounding the teeth to be sampled will be gently removed and dried with cotton gauze. The teeth will then be partially isolated using cotton rolls and a saliva ejector. Additionally, petroleum jelly will be applied to the gingiva for additional protection. A 2-mm-wide microbrush will be rubbed on the carious lesion for 4 s to collect the baseline microbiological sample [3]. The microbrush will then be inserted in a sterile test tube containing 1 mL of saline and transported to the microbiology laboratory within 1–2 h [37]. After collection of the baseline samples, the intervention treatments will be applied as follows:

i. Intervention group (NSF) [17]
  A drop of NSF will be dispensed into a sterile plastic dappen dish.
  Affected tooth surfaces will be dried with a gentle flow of air.
  The solution will be applied to the carious lesions using a microbrush.
  The NSF solution will be left in contact with the tooth surface for 1 min.
  Any excess will be removed with a cotton pellet to minimize systemic absorption.
ii. Control group (SDF) [38]
  A drop of 38% SDF will be dispensed into a sterile plastic dappen dish.
  Affected tooth surfaces will be dried with a gentle flow of air.
  A microbrush will be dipped into the SDF and dabbed on the side of the dappen dish to remove excess liquid before applying the solution to only the affected tooth surface.
  The SDF will be allowed to dry on the lesion for 1 min.
  Any excess will be removed with a cotton pellet to minimize systemic absorption.

*After the application of the fluoride agents*, the children will be instructed not to drink or eat for 1 h after the appointment and their parents will be asked to observe this instruction [23].

To promote sample retention several measures will be taken. Firstly, the participants and their guardians will be provided a thorough explanation of oral hygiene instructions and all their oral health inquires will be addressed to establish rapport between the investigator and the participants at the baseline visit. After the application of the intervention, the participants will be followed up by phone call to monitor for any adverse events that may have occurred and to follow-up with any participant inquires. At the 1 month follow-up appointment, the children will be presented with a small toy/symbolic gift to increase rapport. Finally and most importantly, teeth not treated with SDF or NSF, or those showing signs of failure of caries arrest, or those showing signs of pulpal pathology will be appropriately treated following the standard of care.

##### Follow-up examination

All patients will be recalled for follow-ups after 1 and 3 months. On the day of the recall appointment, patient preparation will be done as mentioned before and microbiological assessment will be performed and CFU counts will be recorded. Lesion activity will be assessed according to the ICDAS II criteria. Using a blunt ended probe, if the cavity is found to be shiny and feels hard on gentle probing of the dentin, it will be recorded as an inactive lesion. While if it remains soft or leathery on gentle probing, it will be recorded as an active lesion (Appendix 2, Table 2).

### Microbiological procedure

#### Sample dilution

All samples will be dispersed by vortex for 30 s then 10-fold serially diluted using sterile saline. A measure of the dilution will then be used for traditional plate culturing methods [8, 39].

#### Culture

Aliquots of 10 μl of each dilution will be inoculated into freshly prepared Mitis Salivarius agar—a selective culture media for *S. mutans* prepared according to the manufacturer’s instructions (Difco Laboratories Inc, NJ, USA)—and Rogosa agar—a selective culture media for lactobacilli prepared according to the manufacturer’s instructions (Himedia Laboratories, Mumbai, India)—respectively, using a micropipette.

- Mitis Salivarius agar plates will be incubated anaerobically in a CO_2_ incubator (Binder redLINE incubator model RI 115-U) with an atmosphere containing 10% CO_2_ at 37 °C for 72 h to detect *Streptococcus mutans* counts.
- Rogosa agar plates will be incubated aerobically at 37 °C for 48 h to detect *Lactobacilli* count.

#### Isolation and enumeration

Following the predetermined incubation period, colonies grown on the specified media will be counted and represented as colony-forming unit (CFU)/mL. *Streptococcus mutans* will be identified based on their characteristic morphology on Mitis Salivarius agar plates. Similarly, *Lactobacilli* will be identified biochemically and microscopically based on their morphology.

The number of colonies will be determined and expressed as colony-forming units using the following equation: [40]

$$\mathrm{CFU}/\mathrm{mL}=\mathrm{no}\ \mathrm{of}\ \mathrm{colonies}\times \mathrm{dilution}\ \mathrm{factor}/\mathrm{volume}\ \mathrm{taken}\ \mathrm{in}\ \mathrm{mL}.$$

### Statistical analysis

The statistical software IBM SPSS Statistics for Windows (Version 25.0. Armonk, NY: IBM Corp) will be used for data analysis. The outcome variable is the number of CFU of *streptococcus mutans* and *lactobacilli*, and the number of arrested caries lesions after 1 and 3 months in both the SDF and NSF groups. The collected data will be recorded and statistically evaluated. As regards the change in microbiological analysis, it will be analyzed using repeated measures ANOVA or Freidman’s test, followed by the suitable post hoc test and as per your valuable recommendation the Bonferroni adjustment will be used. Regarding the change in caries activity, Cochran’s Q test will be used. Regression analysis will be used to assess the potential confounders such as gender, social status, and dietary habits. In order to include all randomized participants in the intention-to-treat analysis, multiple imputation will be performed to impute missing caries activity data and bacterial colony-forming unit log count using all caries indictor variables that will be measured and collected using the WHO questionnaire for oral health assessment of children, these include age, socio-economic status, fluoride exposure, sugar intake, and treatment group. The pooled data of five drawn imputations will be analyzed using Rubin’s rules [41] to produce the adjusted estimates and statistics from which inferences will be drawn. The data will be graphically presented with suitable graphs. The level of statistical significance will be set at 5%. In the case of sample drop out and non-adherence, the intention-to-treat analysis will be employed to account for the missing outcome values in the follow-up appointments.

#### Study outcomes

Treatment effect will be evaluated after 1 and 3 months by comparing:

- Microbiological assessment of CFU of *Streptococcus mutans* and *Lactobacilli* in lesion samples.
- Microbiological assessment of CFU of *Streptococcus mutans* and *Lactobacilli* in saliva samples.
- Caries activity according to ICDAS II criteria.

## Discussion

The use of SDF has been greatly popularized in the last years after its FDA approval in 2014 [42]. However, due to the permanent unesthetic black stain and possibility of soft tissue injury, it is sometimes unacceptable to patients especially for the treatment of anterior teeth [43]. Additionally, soft tissue burns have been reported after the use of SDF [3]. The incorporation of nanotechnology into modern pediatric dentistry may pave the way for a more efficient minimally invasive caries treatment. NSF is a promising cariostatic agent with a potent antibacterial activity and does not produce a black stain [14, 44]. Nanosilver particles have been widely used in a range of medical procedures ranging from treatment [45] and drug delivery [46], to medical device coating [47] and wound dressings [48]. The use of nanosilver products is continually increasing and their incorporation into the dental field holds a promising future. Especially, with the rise of the COVID-19 pandemic, the need for effective minimally invasive dental treatments is of paramount importance. Many dental institutions are not adequately equipped to face the widely spreading virus [49], and the implementation of non-aerosol generating dental procedures is of vital to limit the spread of the virus.

It is important to ensure that the nanosilver product in use is safe and poses no health risks as there have been some concerns related to the cytotoxicity of the nanosilver used [50, 51]. Previous clinical trials using NSF intraorally have synthesized AgNPs using sodium borohydride as a reducing agent [10, 52]. Sodium borohydride is known to be a highly toxic and corrosive chemical that is commonly used in bleaching wood, and its fumes are known to be odorless and cause severe irritation to the lungs and skin. Its use poses a recognized health hazard [53, 54]. For the obvious safety concerns, an alternative method of nanosilver synthesis for clinical investigations would be more appropriate. Therefore, trisodium citrate—an FDA-approved salt widely used in personal and dental care products— [55, 56] was used as a reducing agent instead of sodium borohydride. Additionally, the synthesized AgNPs were PEGylated to increase the stability of the nanosuspension and to prevent particle aggregation. Polyethylene glycol is a popular FDA-approved polymer used extensively as a pharmaceutical stabilizing agent [57, 58]. Characterization tests and cytotoxicity assays are important to ensure the safety of the synthesized formulation for clinical use.

The determination of the suitability of nanosilver fluoride as an anticaries agent and as a valid alternative to SDF is a multifaceted feat. While previous studies have investigated its effect on caries activity [17, 18] and antibacterial potential [12–14], there still remains a lack in the knowledge surrounding its clinical performance as an antibacterial agent. This clinical trial aims to bridge the gap in the available literature on the clinical antibacterial effect of nanosilver fluoride in caries of the primary dentition.

### Ethics approval and consent to participate

This study protocol received approval from the Ethics Committee of the Faculty of Dentistry, Alexandria University (IORG 0008839 - IRB No. 0359-12/2021). A signed informed consent will be obtained by the principal investigator (NA) from every parent. The informed consent that will be signed by the parents will encompass the data to be collected and its intended analysis and use for the study purpose, and it will thoroughly explain the trial procedures, benefits, possible harms/risks, and outline the measures taken to ensure the privacy and confidentiality of the participants. Additionally, the informed consent will include a picture of the black stain caused by SDF treatment. Carious teeth not treated with SDF or NSF, or those showing signs of failure after treatment, or new carious lesions that will appear during the follow-up period, will be appropriately treated. The voluntary nature of the study will be emphasized, and parents will be informed of their right to withdraw from the trial at any time without incurring any penalties.

### Data monitoring

The participants will be contacted by phone after 48 h of the intervention to monitor any reported adverse events or any other unintended effects; if more than half of the participants report adverse events, such as soft tissue injury or pain, the study will be terminated. Furthermore, an interim analysis of the change in microbiological count and lesion activity of the sampled lesions will be conducted at the 1-month follow-up appointment. The study will be terminated if the majority of the treated teeth show signs of continued caries lesion activity as defined by the International Detection and Assessment System (ICDAS II). Active caries lesions will be detected by visual-tactile inspection to assess lesion severity and activity, where if the cavity is found to be shiny and feels hard on gentle probing of the dentin, it is considered an active lesion. Additionally, if the majority of the lesions show signs of caries progression and/or pulpal pathology, the study will be immediately terminated, and the participants will receive the needed restorative treatment. Emphasis will be made on the fact that participants are free to withdraw from the study at any time. No data monitoring committee (DMC) is available at the researcher’s institution and therefore no external body outside of the study team will monitor the data or have access to it.

### Harms

Participants will be contacted by phone after 48 h of the intervention and then monthly for the duration of the trial to assess reported adverse events or any other unintended effects. This will also be assessed at the 1- and 3-month follow-up appointments.

### Biological samples

All collected biological samples (lesion and saliva samples) and associated participant-related data will be coded to protect participant confidentiality. Each participant will be provided with a serial number that will only be accessible to the principal investigator. The biological samples will be cultivated within 1–2 h of collection; upon determining the CFU counts for each sample, the sample will be safely and permanently disposed of. No samples will be stored in any way for future use or for use in any ancillary research.

### Trial status

This is the original version of the protocol, issued in January 2022. The recruitment phase is planned to start in March 2022 and end approximately by May 2022. Any changes or protocol amendments will be accounted for in the public study record available on clinical trials.gov ID: NCT05221749.

### Confidentiality

Data entry will be completed by the principal investigator (NA). The principal investigator cannot be blinded to the interventions as the SDF will inevitably stain the caries lesion black while the NSF is not expected to cause any color change. The principal investigator will also be coding the participants, where each participant will be given a unique serial number that will be stored in a secured file only accessible to the principal investigator. This file will not be shared with anyone and will be stored in a password-protected online drive. Identification numbers will ensure participant confidentiality during data collection and analysis. Only this code/identifier number will be used to identify participants’ samples during microbiological laboratory procedures and during statistical analysis of the results. No biological samples of any participant will be stored for further use after the completion of the microbiological assessment.

### Ancillary and post-trial care

Carious teeth not treated with SDF or NSF, or those showing signs of failure after treatment, or new carious lesions that will appear during the follow-up period, will be appropriately treated.

## Supplementary Information

**Additional file 1: Appendix 1**. Informed Consent Form to Participate in a Research Study. **Appendix 2**. **Table 1**: ICDAS II severity criteria [59]. **Table 2**: ICDAS II caries activity criteria [59]. **Appendix 3**. WHO questionnaire for oral health assessment of children [34].

## Data Availability

The final data set for the proposed study will be available upon reasonable request from the corresponding author.

## References

[1] Kassebaum NJ, Smith AGC, Bernabé E, Fleming TD, Reynolds AE, Vos T (2017). Global, Regional, and national prevalence, incidence, and disability-adjusted life years for oral conditions for 195 countries, 1990-2015: a systematic analysis for the global burden of diseases, injuries, and risk factors. J Dent Res.

[2] Gao SS, Zhang S, Mei ML, Lo ECM, Chu CH (2016). Caries remineralisation and arresting effect in children by professionally applied fluoride treatment - a systematic review. BMC Oral Health.

[3] Milgrom P, Horst JA, Ludwig S, Rothen M, Chaffee BW, Lyalina S (2018). Topical silver diamine fluoride for dental caries arrest in preschool children: A randomized controlled trial and microbiological analysis of caries associated microbes and resistance gene expression. J Dent.

[4] Gao SS, Chen KJ, Duangthip D, Wong MCM, Lo ECM, Chu CH. Arresting early childhood caries using silver and fluoride products – a randomised trial. J Dent. 2020;103(103522). 10.1016/j.jdent.2020.103522 33166594

[5] Rosenblatt A, Stamford TCM, Niederman R (2009). Silver diamine fluoride: A caries “silver-fluoride bullet.”. J Dent Res.

[6] Jabin Z, Vishnupriya V, Agarwal N, Nasim I, Jain M, Sharma A (2020). Effect of 38% silver diamine fluoride on control of dental caries in primary dentition: a systematic review. J Fam Med Prim Care.

[7] Horst JA, Ellenikiotis H, Committee USCA, Milgrom PM (2016). UCSF protocol for caries arrest using silver diamine fluoride: rationale, indications, and consent. J Calif Dent Assoc.

[8] El-Allaky HS, Wahba NA, Talaat DM, Zakaria AS (2020). Antimicrobial effect of propolis administered through two different vehicles in high caries risk children: a randomized clinical trial. J Clin Pediatr Dent.

[9] Frencken JE, Eden E (2016). Dental caries and caries epidemiology. Evidence-based caries prevention.

[10] Targino AGR, Flores MAP, Dos Santos VE, De Godoy Bené Bezerra F, De Luna Freire H, Galembeck A (2014). An innovative approach to treating dental decay in children. A new anti-caries agent. J Mater Sci Mater Med.

[11] Durán N, Silveira CP, Durán M, Martinez DST (2015). Silver nanoparticle protein corona and toxicity: a mini-review. J Nanobiotechnol.

[12] Sayed M, Tsuda Y, Matin K, Abdou A, Martin K, Burrow MF, et al. Effects of mechanical abrasion challenge on sound and demineralized dentin surfaces treated with SDF. Sci Rep. 2020;10(1) [cited 2021 Sep 27]. Available from: 10.1038/s41598-020-77035-9. 10.1038/s41598-020-77035-9 PMC7669835 33199833

[13] Teixeira JA, Costa E, Silva AV, Dos Santos VE, De Melo PC, Arnaud M, et al. Effects of a new nano-silver fluoride-containing dentifrice on demineralization of enamel and Streptococcus mutans adhesion and acidogenicity. Int J Dent. 2018;2018 [cited 2021 Sep 27]. Available from: https://pubmed.ncbi.nlm.nih.gov/29853891/. 10.1155/2018/1351925 PMC5964412 29853891

[14] Vieira Costa E Silva A, Teixeira JA, Mota CCBO, Clayton Cabral Correia Lins E, Correia De Melo P, De Souza Lima MG (2018). In vitro morphological, optical and microbiological evaluation of nanosilver fluoride in the remineralization of deciduous teeth enamel. Nanotechnol Rev.

[15] Yin IX, Zhang J, Zhao IS, Mei ML, Li Q, Chu CH (2020). The antibacterial mechanism of silver nanoparticles and its application in dentistry. Int J Nanomedicine.

[16] Haghgoo R, Saderi H, Eskandari M, Haghshenas H, Rezvani M (2014). Evaluation of the antimicrobial effect of conventional and nanosilver-containing varnishes on oral streptococci. J Dent (Shiraz, Iran).

[17] Tirupathi S, Nirmala SVSG, Rajasekhar S, Nuvvula S (2019). Comparative cariostatic efficacy of a novel nano-silver fluoride varnish with 38% silver diamine fluoride varnish a double-blind randomized clinical trial. J Clin Exp Dent.

[18] Puppala N, Nagireddy VR, Reddy D, Kondamadugu S, Mareddy A, Chris A (2019). Nanosilver fluoride—a paradigm shift for arrest in dental caries in primary teeth of schoolchildren: a randomized controlled clinical trial. Int J Clin Pediatr Dent.

[19] Schulz KF, Altman DG, Moher D (2010). CONSORT 2010 Statement: updated guidelines for reporting parallel group randomised trials. BMC Med.

[20] Rosner B (2015). Hypothesis Testing: Two-Sample Inference. Fundamentals of biostatistics.

[21] Faul F, Erdfelder E. G*Power for Windows (version 3.1.9.7). G*Power. Düsseldorf: Universität Düsseldorf. [cited 2021 Oct 2]. Available from: https://www.psychologie.hhu.de/arbeitsgruppen/allgemeine-psychologie-und-arbeitspsychologie/gpower

[22] Ismail AI, Sohn W, Tellez M, Amaya A, Sen A, Hasson H (2007). The International Caries Detection and Assessment System (ICDAS): an integrated system for measuring dental caries: Methods. Community Dent Oral Epidemiol.

[23] Mitwalli H, Mourao MDA, Dennison J, Yaman P, Paster BJ, Fontana M (2019). Effect of silver diamine fluoride treatment on microbial profiles of plaque biofilms from root/cervical caries lesions. Caries Res.

[24] Agency for Toxic Substances and Disease Registry (2005). Toxicological profiles.

[25] Haahr M (2012). True Random Number Service.

[26] Schulz KF, Grimes DA (2002). Allocation concealment in randomised trials: defending against deciphering. Lancet..

[27] International Caries Classification and Management System (ICCMS). ICDAS Foundation. ICDAS elearning courses. Available from: https://www.iccms-web.com/content/iccms-usage/clinical-practice. Cited 2021 Oct 14.

[28] American Academy of Pediatric dentistry (2017). Chairside guide: Silver diamine fluoride in the management of dental caries lesions. Pediatr Dent.

[29] Gakiya-Teruya M, Palomino-Marcelo L, Rodriguez-Reyes JCF (2019). Synthesis of highly concentrated suspensions of silver nanoparticles by two versions of the chemical reduction method. Methods Protoc.

[30] Raouf M, Essa S, El Achy S, Essawy M, Rafik S, Baddour M (2021). Evaluation of combined ciprofloxacin and azithromycin free and nano formulations to control biofilm producing Pseudomonas aeruginosa isolated from burn wounds. Indian J Med Microbiol.

[31] Essawy MM, El-Sheikh SM, Raslan HS, Ramadan HS, Kang B, Talaat IM (2021). Function of gold nanoparticles in oral cancer beyond drug delivery: implications in cell apoptosis. Oral Dis.

[32] Jiménez-Ramírez AJ, Martínez-Martínez RE, Ayala-Herrera JL, Zaragoza-Contreras EA, Domínguez-Pérez RA, Reyes-López SY, et al. Antimicrobial Activity of Silver Nanoparticles against Clinical Biofilms from Patients with and without Dental Caries. J Nanomater. 2021;2021:13. Article ID 5587455. 10.1155/2021/5587455.

[33] Weinstein MP (2021). M100 performance standards for antimicrobial susceptibility testing.

[34] Petersen PE, Baez RJ, Organization WH (1977). Oral health surveys; basic methods.

[35] Çağlar E, Kuşcu OO (2016). The role of diet in caries prevention. Evid-Based Caries Prev.

[36] Kamate WI, Vibhute NA, Baad RK (2017). Estimation of DMFT, salivary streptococcus mutans count, flow rate, Ph, and salivary total calcium content in pregnant and non-pregnant women: A prospective study. J Clin Diagn Res.

[37] Rishmawi N, Ghneim R, Kattan R, Ghneim R, Zoughbi M, Abu-Diab A (2007). Survival of fastidious and nonfastidious aerobic bacteria in three bacterial transport swab systems. J Clin Microbiol.

[38] Elevate Oral Care. Safety data sheet Safety data sheet, advantage arrest 38 % silver diamine fluoride. 2005 1173.. p. 1–8. [cited 2021 Sep 28]. Available from: http://www.elevateoralcare.com/dentist/AdvantageArrest/Advantage-Arrest-Silver-Diamine-Fluoride-38

[39] Karched M, Ali D, Ngo H (2019). In vivo antimicrobial activity of silver diammine fluoride on carious lesions in dentin. J Oral Sci.

[40] Ravindran S, Chaudhary M, Gawande M (2013). Enumeration of salivary Streptococci and Lactobacilli in children with differing caries experiences in a rural Indian population. ISRN Plast Surg.

[41] Rubin DB. Multiple imputation for nonresponse in surveys. Wiley. 2004; [cited 2022 Jun 5]. Available from: https://www.wiley.com/en-us/Multiple+Imputation+for+Nonresponse+in+Surveys-p-9780471655749.

[42] U.S Food & Drug Administration (2014). Diammine silver fluoride dental hypersensitivity varnish.

[43] Sabbagh H, Othman M, Khogeer L, Al-Harbi H, Al Harthi A, Abdulgader Yaseen Abdulgader A (2020). Parental acceptance of silver diamine fluoride application on primary dentition: a systematic review and meta-analysis. BMC Oral Health.

[44] Espíndola-Castro LF, Rosenblatt A, Galembeck A, de Melo Monteiro GQ (2020). Dentin staining caused by nano-silver fluoride: a comparative study. Oper Dent.

[45] Sibbald RG, Contreras-Ruiz J, Coutts P, Fierheller M, Rothman A, Woo K (2007). Bacteriology, inflammation, and healing: a study of nanocrystalline silver dressings in chronic venous leg ulcers. Adv Skin Wound Care.

[46] Skirtach AG, Muñoz Javier A, Kreft O, Köhler K, Piera Alberola A, Möhwald H (2006). Laser-induced release of encapsulated materials inside living cells. Angew Chem Int Ed Engl.

[47] Galiano K, Pleifer C, Engelhardt K, Brössner G, Lackner P, Huck C (2008). Silver segregation and bacterial growth of intraventricular catheters impregnated with silver nanoparticles in cerebrospinal fluid drainages. Neurol Res.

[48] Moore K (2006). A new silver dressing for wounds with delayed healing Product REVIEW KEY WORDS Atrauman Ag Chronic wound Critical colonisation Wound infection Silver dressing. Wounds UK.

[49] Ammar N, Aly NM, Folayan MO, Khader Y, Mohebbi SZ, Attia S (2021). Perceived preparedness of dental academic institutions to cope with the covid-19 pandemic: a multi-country survey. Int J Environ Res Public Health.

[50] Burns J, Hollands K (2015). Nano Silver Fluoride for preventing caries. Evid Based Dent.

[51] Ferdous Z, Nemmar A. Health impact of silver nanoparticles: a review of the biodistribution and toxicity following various routes of exposure. Int J Mol Sci. 2020;21(7) [cited 2022 Jan 24]. Available from: /pmc/articles/PMC7177798/. 10.3390/ijms21072375 PMC7177798 32235542

[52] Dos Santos VE, Filho AV, Ribeiro Targino AG, Pelagio Flores MA, Galembeck A, Caldas AF (2014). A new “silver-bullet” to treat caries in children – nano silver fluoride: a randomised clinical trial. J Dent.

[53] National Library of Medicine, National Center for Biotechnology Information. Sodium borohydride | BH4.Na - PubChem. [cited 2021 Dec 17]. Available from: https://pubchem.ncbi.nlm.nih.gov/compound/Sodium-borohydride

[54] New Jersey Department of Health and Senior Services (2007). Hazardous Substances Fact Sheet - Sodium Borohydride.

[55] U.S. Food & Drug Administration (1987). Sodium citrate. CFR - Code of Federal Regulations.

[56] National Library of Medicine, National Center for Biotechnology Information. Trisodium citrate dihydrate | C6H9Na3O9 - PubChem. PubChem - NIH. [cited 2021 Dec 17]. Available from: https://pubchem.ncbi.nlm.nih.gov/compound/Trisodium-citrate-dihydrate

[57] U.S Food and Drug Administration (2021). Drugs@FDA: FDA-approved drugs. Drug Databases.

[58] National Cancer Institute at the National Institutes of Health (2021). NCI Dictionary of Cancer Terms - PEG.

[59] Dikmen B (2015). Icdas Ii Criteria (International Caries Detection and Assessment System). J Istanbul Univ Fac Dent.

