# Supplementary material for: The antibacterial effect of nanosilver fluoride in relation to caries activity in primary teeth: a protocol for a randomized controlled clinical trial
Source: Trials. 2022 Jul 8;23:558. doi: 10.1186/s13063-022-06477-5 (PMC9264752; doi:10.1186/s13063-022-06477-5)
Supplement: Supplementary file 1 — Additional file 1: Appendix 1. Informed Consent Form to Participate in a Research Study. Appendix 2. Table 1: ICDAS II severity criteria [59]. Table 2: ICDAS II caries activity criteria [59]. Appendix 3. WHO questionnaire for oral health assessment of children [34]. [file 13063_2022_6477_MOESM1_ESM.docx]

**APPENDIX I**

**Informed Consent Form to Participate in a Research Study**

**Name of the researcher:** Nour Sherif Ammar

**Title of the research:** The Antibacterial Effect of Nanosilver Fluoride in Relation to Caries Activity in Primary Teeth: A Randomized Controlled Clinical Trial

**Department:** Department of Pediatric Dentistry and Dental Public Health, Faculty of Dentistry, Alexandria University, Alexandria, Egypt.

1. **Purpose of the research:** Evaluation of the antibacterial effect of nanosilver fluoride and silver diamine fluoride on carious lesions in primary teeth.
2. **Number of participants:** 50
3. **Study type:** clinical trial
4. **Duration:** 3 months
5. **Risks:** The risk of black discoloration with SDF treatment.
6. **Benefits:** The participating child will benefit the possibility of arresting the treated active caries lesions and decrease the amount of caries causing bacteria in the child’s mouth.
7. **Confidentiality:** We will not be sharing information about you to anyone outside of the research team. The information that we collect from this research project will be kept private. Any information about you will have a number on it instead of your name.
8. **Methods of treating any injury that may occur from the study:** The researcher will take all measures to prevent any injury or disease that may occur as a result of this study. But if any unexpected injury occurred as a result of your participation in the study, you will receive the necessary treatment at the college.
9. **Biological sample collection:** All collected biological samples and associated participant-related data will be coded to protect confidentiality. Each participant will be provided with a serial number that will only be accessible to the principal investigator. The biological samples will be processed within 1-2 hours of collection, upon determining the bacterial counts for each sample the sample will be safely and permanently disposed of. No samples will be stored in any way for future use or for use in any other research purposes.
10. **Reimbursements:** You will not be provided any incentive to take part in the research.
11. **Right to refuse or withdraw:** You do not have to take part in this research if you do not wish to do so. You may stop participating in the study at any time that you wish.
12. **If you have any questions**, you can ask them now or later. If you wish to ask questions later, you may contact: [Nour Ammar: 034868066, nour.ammar@alexu.edu.eg] or report any problem to Dr. Hassan Kassem, ethics committee coordinator: 03486969.
13. **Statement by the researcher/person taking consent**

I confirm that the participant was given an opportunity to ask questions about the study, and all the questions asked by the participant have been answered correctly and to the best of my ability. I confirm that the individual has not been coerced into giving consent, and the consent has been given freely and voluntarily.

A copy of this informed consent form has been provided to the participant.

Print Name of Researcher/person taking the consent________Nour Ammar_______________

Signature of Researcher /person taking the consent___________________________________

Date ________________________________________________________ (Day/month/year)

1. Certificate of Consent

I have read the foregoing information, or it has been read to me. I have had the opportunity to ask questions about it and my questions have been answered to my satisfaction. I consent voluntarily to be a participant in this study.

Print Name of Participant_____________________________________________________

Signature of Participant ______________________________________________________

Date _______________________________________________________ (Day/month/year)

**APPENDIX II**

**Table (1): ICDAS II severity criteria**(59)

| **Score** | **Criteria** |
| --- | --- |
| **0** | No change in enamel translucency with prolonged air drying |
| **1** | First visual change in enamel (after prolonged air drying) |
| **2** | Distinct visual change in enamel |
| **3** | Localized enamel breakdown or discolored enamel with no visible dentine involvement |
| **4** | Underlying dark shadow from dentine |
| **5** | Distinct cavity with visible dentine |
| **6** | Extensive distinct cavity with visible dentine |

**APPENDIX II**

**Table (2): ICDAS II caries activity criteria**(59)

| **ICDAS code** | **Characteristics of Lesion** | |
| --- | --- | --- |
|  | **Active Lesion** | **Inactive Lesion** |
| **1, 2 or 3** | Surface of enamel is whitish/yellowish opaque with loss of luster; feels rough when the tip of the probe is moved. Lesion is in a plaque stagnation area, i.e.: pits and fissures, near the gingival and approximal surface below the contact point. | Surface of enamel is whitish, brownish or black. Enamel may be shiny and feels hard and smooth when the tip of the probe is moved. For smooth surfaces, caries lesion is typically located at some distance from the gingival margin |
| **4** | Probably active | |
| **5 or 6** | Cavity feels soft or leathery on gently probing the dentin | Cavity may be shiny and feels hard on gently probing the dentin. |

**APPENDIX III**

**WHO questionnaire for oral health assessment of children** (33)

| ***First, we would like you to answer some questions concerning yourself and your teeth*** |
| --- |
| **Identification number Sex Location**  Boy Girl Urban Periurban Rural  1. ☐☐☐☐ ☐ ☐ ☐ ☐ ☐  1 4 1 2 1 2 3 |
| 2. **How old are you today?** (Years) |
| 3. **How would you describe the health of your teeth and gums?**  (Read each item)  **Teeth Gums**  Excellent ................................................................ ☐ 1 ☐ 1  Very good............................................................... ☐ 2 ☐ 2  Good ..................................................................... ☐ 3 ☐ 3  Average .................................................................. ☐ 4 ☐ 4  Poor ....................................................................... ☐ 5 ☐ 5  Very poor ............................................................... ☐ 6 ☐ 6  Don’t know ............................................................ ☐ 9 ☐ 9 |
| 4. **How often during the past 12 months did you have toothache or feel discomfort due to your teeth?**  Often ☐ 1  Occasionally ☐ 2  Rarely ☐ 3  Never ☐ 4  Don’t know ☐ 9 |
| ***Now please answer some questions about the care of your teeth*** |
| 5. **How often did you go to the dentist during the past 12 months?**  (Put a tick/cross in one only)  Once ☐ 1  Twice ☐ 2  Three times ☐ 3  Four times ☐ 4 |

| More than four times ☐ 5  I had no visit to dentist during the past 12 months ☐ 6  I have never received dental care/visited a dentist ☐ 7  I don’t know/don’t remember ☐ 9 |
| --- |
| ***If you did not see a dentist during the last 12 months, go on to question 7*** |
| 6. **What was the reason for your last visit to the dentist?**  (Put a tick/cross in one box only)  Pain or trouble with teeth, gums or mouth ☐ 1  Treatment/follow-up treatment ☐ 2  Routine check-up of teeth/treatment ☐ 3  I don’t know/don’t remember ☐ 9 |
| 7. **How often do you clean your teeth?**  (Put a tick/cross in one box only) Never ☐ 1  Several times a month (2–3 times) ☐ 2  Once a week ☐ 3  Several times a week (2–6 times) ☐ 4  Once a day ☐ 5  2 or more times a day ☐ 6 |
| 8. **Do you use any of the following to clean your teeth or gums?**  (Read each item)  Yes No  1 2  Toothbrush............................................................. ☐ ☐  Wooden toothpicks ................................................. ☐ ☐  Plastic toothpicks.................................................... ☐ ☐  Thread (dental floss) .............................................. ☐ ☐  Charcoal ................................................................ ☐ ☐  Chewstick/miswak................................................... ☐ ☐  Other ..................................................................... ☐ ☐  Please specify |
| 1. Yes No    1. **Do you use toothpaste to clean your teeth?**.......... ☐ 1 ☐ 2   Yes No   - 1. **Do you use toothpaste that contains fluoride?**.... ☐ 1 ☐ 2   Don’t know ☐ 9 |

| 1. **Because of the state of your teeth and mouth, have you experienced any of the following problems during the past year?**   Yes No Don’t know  1 2 0   - 1. I am not satisfied with the   appearance of my teeth .................. ☐ ☐ ☐   - 1. I often avoid smiling and laughing   because of my teeth ....................... ☐ ☐ ☐   - 1. Other children make fun of   my teeth ........................................ ☐ ☐ ☐   - 1. Toothache or discomfort caused by my teeth forced me to miss classes at school or miss school   for whole days................................ ☐ ☐ ☐   - 1. I have difficulty biting hard foods ...... ☐ ☐ ☐   2. I have difficulty in chewing................... ☐ ☐ ☐ |
| --- |
| 11. **How often do you eat or drink any of the following foods, even in small quantities?**  (Read each item)  Several Several Several times Every times Once times  a day day a week a week a month Never  6 5 4 3 2 1  Fresh fruit................... ☐ ☐ ☐ ☐ ☐ ☐  Biscuits, cakes, cream cakes, sweet pies,  buns etc................... ☐ ☐ ☐ ☐ ☐ ☐  Lemonade, Coca Cola  or other soft drinks .. ☐ ☐ ☐ ☐ ☐ ☐  Jam/honey ................... ☐ ☐ ☐ ☐ ☐ ☐  Chewing gum  containing sugar ...... ☐ ☐ ☐ ☐ ☐ ☐  Sweets/candy............... ☐ ☐ ☐ ☐ ☐ ☐  Milk with sugar ........... ☐ ☐ ☐ ☐ ☐ ☐  Tea with sugar ............ ☐ ☐ ☐ ☐ ☐ ☐  Coffee with sugar . . . .. ☐ ☐ ☐ ☐ ☐ ☐  **(Insert country-specific items)** |

| 12. **How often do you use any of the following types of tobacco?**  (Read each item)  Several Several Every times Once times  day a week a week a month Seldom Never  6 5 4 3 2 1  Cigarettes, pipe or cigars ... ☐ ☐ ☐ ☐ ☐ ☐  Chewing tobacco or snuff .. ☐ ☐ ☐ ☐ ☐ ☐ |
| --- |
| 13. **What level of education has your father completed (or your stepfather, guardian or other male adult living with you)?** No formal schooling ☐ 1  Less than primary school ☐ 2  Primary school completed ☐ 3  Secondary school completed ☐ 4  High school completed ☐ 5  College/university completed ☐ 6  No male adult in household ☐ 7  Don’t know ☐ 9 |
| 14. **What level of education has your mother completed?**  No formal schooling ☐ 1  Less than primary school ☐ 2  Primary school completed ☐ 3  Secondary school completed ☐ 4  High school completed ☐ 5  College/university completed ☐ 6  No female adult in household ☐ 7  Don’t know ☐ 9  **(Insert country-specific categories)** |
| ***That completes our questionnaire***  ***Thank you very much for your cooperation!*** |
| Year Month Day Interviewer District Country  ☐☐ ☐☐ ☐☐ ☐☐ ☐☐ ☐☐☐☐ |
